# Feasibility of Using Rice Leaves Hyperspectral Data to Estimate CaCl_2_-extractable Concentrations of Heavy Metals in Agricultural Soil

**DOI:** 10.1038/s41598-019-52503-z

**Published:** 2019-11-06

**Authors:** Weihong Zhou, Jingjing Zhang, Mengmeng Zou, Xiaoqing Liu, Xiaolong Du, Qian Wang, Yangyang Liu, Ying Liu, Jianlong Li

**Affiliations:** 1 School of Life Sciences, Nanjing University, Nanjing, 210093 China; 2 Suzhou Institute of Technology, Jiangsu University of Science and Technology, Zhangjiagang, 215600 China

**Keywords:** Environmental monitoring, Environmental impact

## Abstract

Heavy metals contamination is a serious problem of China. It is necessary to estimate bioavailability concentrations of heavy metals in agricultural soil for keeping the food security and human health. This study aimed to use hyperspectral data of rice (*Oryza sativa*) leaves as an indicator to retrieve the CaCl_2_-extractable concentrations of heavy metals in agricultural soil. Twenty-one rice samples, soil samples and reflectance spectra of rice leaves were collected, respectively. The potential relations between hyperspectral data and CaCl_2_-extractable heavy metals (E-HM) were explored. The partial least-squares regression (PLSR) method with leave-one-out cross-validation has been used to predict concentrations of CaCl_2_-extractable cadmium (E-Cd) and concentrations of CaCl_2_-extractable lead (E-Pb) in farmland soil. The results showed that the concentrations of E-Cd in soil had significant correlation with concentrations of Cd in rice leaves; the number of bands associated with E-Cd was more than that of E-Pb. Four indices (normalized difference vegetation index (NDVI), carotenoid reflectance index (CRI), photochemical reflectance index 2 (PRI2), normalized pigments chlorophyll ratio index (NPCI)) were significant (*P* < 0.05) and negatively related to the E-Cd concentrations. The PLSR model of E-Cd concentrations performed better than the PLSR model of E-Pb concentrations, which with *R*^2^ = 0.592 and RMSE = 0.046. We conclude that if the rice was sensitive to E-HM and/or the crop was stressed by the E-HM, the hyperspectral data of field rice leaves hold potentials in estimating concentration of E-HM in farmland soil. Therefore, this method provides a new insight to monitoring the E-HM content in agricultural soil.

## Introduction

Heavy metals in agricultural soil are very persistent, they do not be biodegrade and they readily accumulate to toxic levels^1^. In general, heavy metals can migrate from polluted soil and/or irrigation water to vegetables and crops, leading, after chronic consumption, to food security and to health problems^2^. Therefore, the situation of soil especially agricultural soil heavy metal pollution in the farmland soil cannot be ignored.

Rice is the most widely consumed cereal grain on earth, the global rice production was over 740 million tonnes in 2014, with Asian countries, including China, Thailand, Japan, and Indonesia dominating the global rice production^3^. Rice cultivated in the polluted paddy soil area can affect human health detrimentally^4^. It has been reported that the soils in China polluted by heavy metals alone account for almost one-sixth of the total cultivated land and those polluted soils are mainly distributed in the intensively cultivated areas^5^, so, many rice are still cultivated in the large-scale slightly and moderately heavy metal contaminated soil^6^.

Given the concerns of monitoring heavy metals in farmland, numerous research efforts have been conducted to assess the total amount of heavy metals in farmland soil^7,8^. But currently researchers realized that the total metal content in the solid phase often does not well predict toxic effects in soil dwelling organisms and plants^9–11^. Instead, organisms respond only to the fraction that is biologically available for that organism^11^. In the last few decades, researchers have followed different extraction techniques to estimate the fractionation of metals in soil/ sediments^12–14^. 0.01 M CaCl_2_ is a commonly used selective chemical extractant^15–17^, because 0.01 M CaCl_2_ solution matches the soil solution with respect to pH, concentration and composition^18^. Novozamsky *et al*. (1993) reported that a close relationship was found between the Cd concentration in vegetables and its concentration in the CaCl_2_-extract^19^. Anjos *et al*.(2012) use five extractant solutions evaluate the available fraction of aluminium (Al), Pb, manganese (Mn) and zinc (Zn) in the Pb mine, and found that CaCl_2_ seems to be a good extractant medium^20^.

However, traditional CaCl_2_-extract methods is time-consuming and expensive^21^. And it is highly challenging to use the field sampling and wet chemistry methods for regular monitoring of heavy metal uptake at large scales. Compared with most chemical analysis, remote sensing technology has the advantages of simple, time-saving and labor-saving in soil monitoring^22^, especially the emergence of hyperspectral remote sensing technology makes it possible to monitor soil minerals, water, nutrients, salinity and other elements. With the continuous sampling and the high spectral resolution (<5 nm), hyperspectral sensors can discriminate critical spectral differentials in detail^23^. Some researchers have applied hyperspectral reflectance to detect the heavy metal in soil^8,24,25^. However, in the present study, some heavy metals are spectrally featureless in the visible and near-infrared parts of the electromagnetic spectrum^26^.

Compared with straightforward to derive heavy metal concentrations in soil, indirect access to soil heavy metal concentrations by plants is more practical. When plants are stressed, the biochemical contents (e.g., chlorophyll) of their leaves may change, and the spectral properties (reflectance and transmittance) at specific wavelengths (e.g., red, green, blue and red edge bands) will change with the biochemical contents of plants leaves^27^. Therefore, plants can be used as bridges to detect the elements in the soil using hyperspectral remote sensing techniques. Hyperspectral remote sensing has been used to detect stress in plants before visible symptoms have been observed^28–30^, such as water deficiency^31^, metal accumulation^32^, diseases^33^ and salt^34^. Compared with monitoring stress in plants, use plant as an indicator to estimating CaCl_2_-extractable concentrations of heavy metals in agricultural soil by use the remote or proximal sensing is less studied and rarely reported in the literature according to our reviews.

The main objective of this study was to evaluate the effectiveness of using spectral reflectance at leaf scales to quantify the heavy metal concentrations in agricultural soil in Zhangjiagang, Suzhou, China. The aims of our study were: (1) to analysis the relationship between heavy metals concentrations in rice leaves and CaCl_2_-extractable heavy metals (E-HM) concentrations in soil; (2) to determine the optimum variables that provide great sensitivity to E-HM concentrations; and (3) to establish the PLSR model for estimating E-HM concentrations in agricultural soil using optimum sensitive variables of hyperspectral data of rice leaves.

## Materials and Methods

### Description of study area

**Figure 1:**
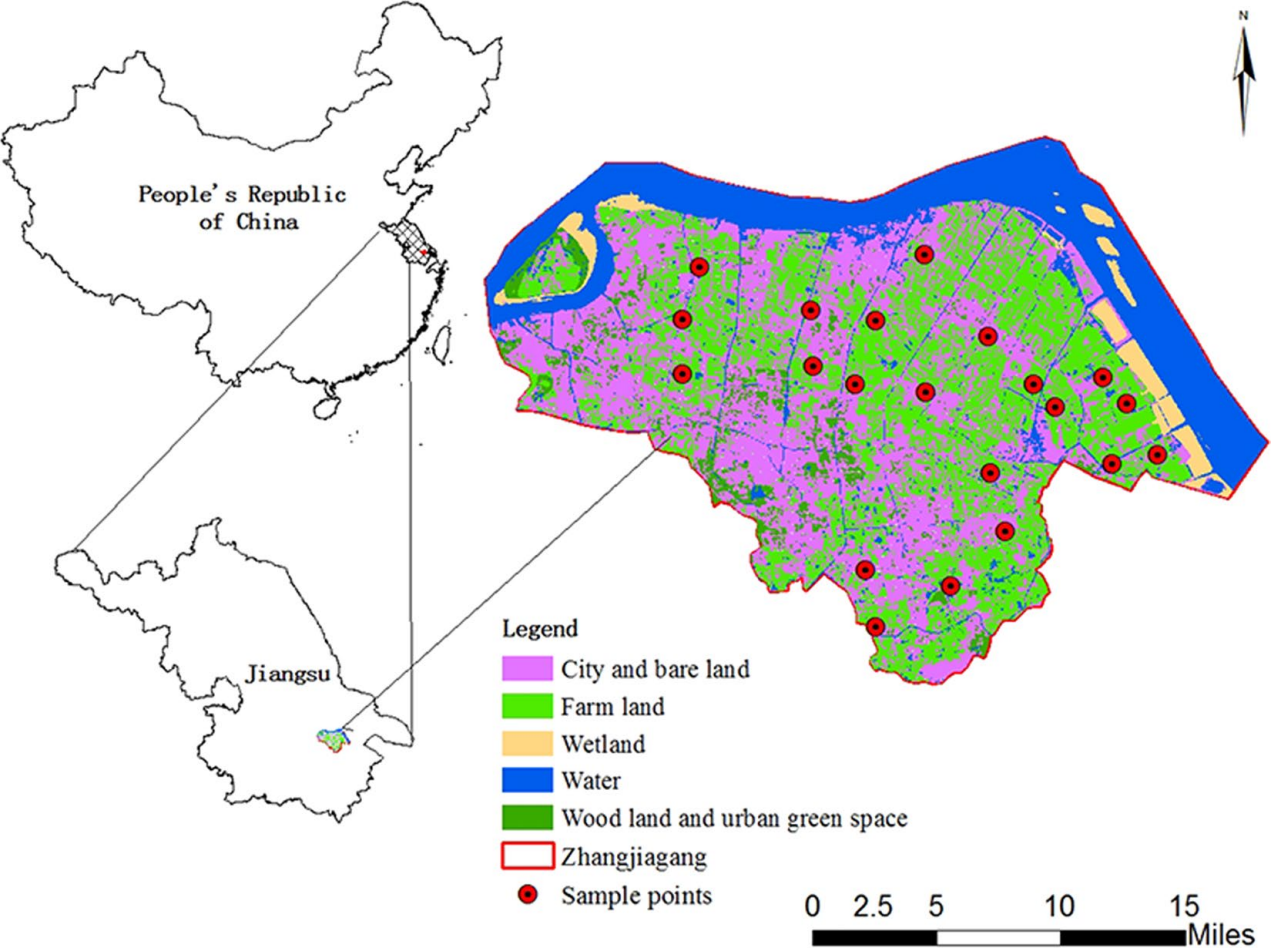
Location of the Zhangjiagang city and field sample sites.

Located on the eastern of the Yangtze River Delta (31°43′-32°02′N, 120°21′-120°52′E), Zhangjiagang city is approximately 999 km^2^, of which 799 km^2^ are terrestrial areas (Fig. 1). The average annual temperature is 17.3 °C and the average annual precipitation is 1556.5 mm^35^. The soil type is mainly fluvo-aquic soil and paddy soil^36^. Because of the developed chemical industry, metallurgy, electroplating industry, printing and dyeing papermaking, *et al*., the Zhangjiagang city is one of the fastest growing cities in the Yangtze River Delta.

### Field sampling and hyperspectral measurement

A total of 21 sampling sites (Fig. 1) were set during September, 2017 in agricultural areas. At each sampling site, hyperspectral reflectance of the rice leaves, samples of rice and their root-soil (0–20 cm depth) were taken. The rice samples and soil were packed into polyethylene bags. Five random samples on each site were taken and bulked together as one composite sample. The location of each sampling site was acquired using a Global Positioning System (GPS, UniStrong G120) with an accuracy of about 3 m.

The hyperspectral reflectance of the rice leaves was obtained using a field portable spectrometer (UniSpec, PP systems, Haverhill, MA, USA). Spectral range and the spectral resolution of the sensor were 310–1100 nm and 3.3 nm, respectively. A bifurcated fiber optic cable and a leaf clip (models UNI410 and UNI501, PP Systems, Haverhill, MA, USA) were used to measure leaf reflectance of rice. The leaf clip held the fiber at a 60° angle to the leaf surface. Leaf illumination was provided by a halogen lamp in the spectrometer through one side of the bifurcated fiber. To minimize the measurement noise of the reflectance spectral, three spectral measurements of the fully-expanded leaves near top of each bundle were made and the 15 results averaged as one spectral measurement for the sampling site. A barium sulfate panel was used as a white reference standard to calibrating and optimizing the spectral before each measurement.

### Laboratory analysis

#### Soil heavy metals concentrations measurements

Soil samples were air-dried at room temperature (26–28 °C), and then sieved through a 2-mm nylon mesh to remove stones or other debris. Total concentrations of Cd and Pb in the soil were determined as following steps: 0.2 g soil was digested with 10 ml mixed solution of HNO_3_, HClO_4_ and HF (1:1:2, v/v/v) in a polytetrafluoroethylene digestion tank, microwave digestion for 15 minutes (according to different sample conditions, the proportion of acid and digestion time can be adjusted), the final solution was diluted to 50 ml using deionized distilled water and analyzed with inductively coupled plasma atomic mass spectrometry (ICP-MS, X2, Thermo Electron Corporation) after digestion by a mixture of concentrated^37,38^.

CaCl_2_-extractable concentrations of Cd and Pb were determined as following steps: a 25 ml aliquot of 0.01 M CaCl_2_ solution was added into a 5 g soil (<2 mm) sample in a 100 ml conical flask and the suspension was shaken at 250 rpm at 25 °C. After 12 h of shaking, the supernatant was separated from the solid phase by centrifugation at 3000 rpm for 20 min. The concentrations of Pb and Cd in the supernatant were analyzed with ICP-MS^18^.

Rice samples were thoroughly washed in deionized water, oven-dried at 70 °C until constant weight. For analyzing Cd and Pb concentration in rice leaves, 0.2 g sample was digested with 5 ml mixed solution of 5:2 HNO_3_: H_2_O_2_ (v/v) in centrifuge tubes at room temperature. Then this solution was heated in a microwave accelerated reaction system (Anton-Paar PE Multiwave 3000) for 20 min. The digested substrate was then diluted with 43 ml deionized water and analyzed for total Cd and Pb with ICP-MS.

### Hyperspectral data pretreatment

The original hyperspectral signal is susceptible to the environment, so original spectra data were preprocessed to enhance the spectral features and to acquire more information about heavy metals in the soil. Wavelengths shorter than 420 nm and longer than 980 nm were not analyzed due to excessive noises^39^, thus a total of 560 wavelengths were used as the raw spectral reflectance and were automatically interpolated from 3.3 nm to 1 nm in calibration^23^. This process was done using Excel 2007 (Microsoft Inc.).

Derivative transformation can remove the interferences of background, resolve overlapping spectra, and minimize the baseline drift of raw spectra that is caused by differences in grinding and optical setups^40^. The first derivative transformation and second derivative transformation were done using OriginPro 8 software.

### Spectral indices calculated

Ten commonly used spectral indices were calculated (Table 1). As shown in Table 1, except for water index (WI), all other spectral indices are related to chlorophyll or pigment.

**Table 1:** Spectral indices used in this study.

Spectral indices name	Abbreviation	Formulation	Reference
1. Normalized difference vegetation index	NDVI	(R_800_ − R_670_)/(R_800_ + R_670_)	^60^
2. Simple ratio index	SR	R750/R705	^61^
3. Vogelmann red edge index	VOGI	R740/R720	^62^
4. Modified simple ratio index	mSR_705_	(R750 − R445)/(R705 − R445)	^63^
5. Anthocyanin reflectance index	ARI	(1/R550) − (1/R700)	^64^
6. Water index	WI	R900/R970	^65^
7. Photochemical reflectance index 2	PRI2	(R570 − R539)/(R570 + R539)	^66^
8. Carotenoid reflectance index	CRI	(1/R510) − (1/R550)	^67^
9. Normalized pigments chlorophyll ratio index	NPCI	(R_680_ − R_460_)/(R_680_ + R_460_)	^68^
10. Red-edge vegetation stress index	RVSI	((R_714_ + R_752_)/2) − R_733_	^69^

R_x_ is the reflectance at x nm.

### Variables selected and partial least-squares regression model built

Correlation analysis of E-HM concentrations with the raw spectral reflectance (R), first-order differential of R (R′), second-order differential of R (R′′) and spectral indices respectively were performed in SPSS (IBM SPSS Statistics 22) using bivariate related analysis. Variables with a significant correlation (*P* < 0.05) were selected for use in the model.

The partial least-squares regression (PLSR) with leave-one-out cross-validation was used to predict E-HM concentrations in farmland soil using selected variables and spectral indices. PLSR is one of the most frequently used methods for the estimation of soil heavy metal concentrations with visible and near-infrared reflectance spectroscopy (VNIRS)^40–42^. It can process data with strong collinearity and noise, and is well suited for situations where the number of variables considerably exceeds the number of available samples^43,44^. The PLSR and cross-validation were performed in TQ Analyst (8.3.125, Thermo Fisher Scientific Inc.). The performances of PLSR were assessed with two evaluation parameters between the measured values and predicted values: the coefficient of determination (*R*^2^) and root mean square error (RMSE). The *R*^2^ and the RMSE are commonly calculated using the following formulas^45^:

1 $${R}^{2}=\frac{{[{\sum }_{{\rm{i}}=1}^{{\rm{n}}}({{\rm{x}}}_{{\rm{p}}}-\overline{{{\rm{x}}}_{{\rm{p}}}}){\sum }_{{\rm{i}}=1}^{{\rm{n}}}({{\rm{x}}}_{{\rm{m}}}-\overline{{{\rm{x}}}_{{\rm{m}}}})]}^{2}}{{\sum }_{{\rm{i}}=1}^{{\rm{n}}}{({{\rm{x}}}_{{\rm{p}}}-\overline{{{\rm{x}}}_{{\rm{p}}}})}^{2}{\sum }_{{\rm{i}}=1}^{{\rm{n}}}{({{\rm{x}}}_{{\rm{m}}}-\overline{{{\rm{x}}}_{{\rm{m}}}})}^{2}}$$

2 $${\rm{RMSE}}=\sqrt{\frac{{\sum }_{{\rm{i}}=1}^{{\rm{n}}}{({{\rm{x}}}_{{\rm{p}}}-{{\rm{x}}}_{{\rm{m}}})}^{2}}{{\rm{n}}}}$$

where x_p_ and $\overline{{{\rm{x}}}_{{\rm{p}}}}\,\,$are the predicted value and the average predicted value of E-HM concentrations, x_m_ and $\overline{{{\rm{x}}}_{{\rm{m}}}}$ are the measured value and the average measured value of E-HM concentrations and n is the number of samples.

## Results and Discussion

### Heavy metal concentrations in agricultural soil

Descriptive statistics of concentration of Cd, Pb were reported in Table 2. It illustrated that the average concentrations of Pb (29.193 mg kg^−1^) was below the limit (80 mg kg^−1^) set by Ministry of Ecology and Environment of the People’s Republic of China (MEEPRC)^46^, while the average concentration of Cd (0.301 mg kg^−1^) may be affected by human activities was slightly higher than the limit (0.3 mg kg^−1^) set by MEEPRC. In addition, the concentration of Cd in four samples (4 out of 21) exceeded the limit set by MEEPRC. Also, the mean concentration of Pb was bigger than the mean concentration of Cd, but the relationship between the mean concentration of E-Pb and E-Cd was reverse. That was because Cd is more available than Pb in soil^47,48^.

**Table 2:** Heavy metal concentrations (mg kg^−1^) of agricultural soil (n = 21) in Zhangjiagang city.

Heavy metal concentrations	Range	Mean	Median	SD	CV %	EN	ER %
Cd	0.110–1.416	0.301	0.209	0.297	98.480	4	19.048
Pb	20.595–57.186	29.193	26.842	8.001	27.407	0	0
E-Cd	0.007–0.257	0.051	0.022	0.0693	135.861	—	—
E-Pb	0.002–0.078	0.01	0.003	0.0173	173.231	—	—

SD, standard deviation; CV, coefficient variation; EN, the number of samples exceeded the limit set by MEEPRC; ER, the rate of samples exceeded the limit set by MEEPRC.

The mean SD and CV of E-Cd concentrations and E-Pb concentrations were also shown in Table 2. The CV of E-Cd concentrations and E-Pb concentrations were different from it of Cd concentrations and Pb concentrations. Forevermore, the soil with high concentrations of Cd and Pb may not have high concentrations of E-Cd and E-Pb. That may because that the E-HM concentrations in natural soils depends on differences soil environment, such as pH, concentrations of clay, sand and organic matter^9^.

### Relationship between heavy metals concentrations in soil and those in rice leaves

The Pearson’s correlation coefficients between heavy metals concentrations in soil and in rice leaves are shown in Table 3. Only the significance of Pearson’s correlation coefficients between E-Cd in soil and Cd in rice leaves was 0.649, which reached to the level of 0.05; While the significance of Pearson’s correlation coefficients between Pb in soil and Pb in rice leaves were 0.340 for concentration of E-Pb and 0.222 for total concentration of Pb. Compared with concentrations of E-Pb, the concentrations of E-Cd had higher correlation with Cd concentrations rice leaves relatively in our study. Earlier studies found that, at common soil pH range, the stability of Cd is lower than that of Pb^49,50^. Meanwhile, rice tends to accumulate Cd, and the accumulation of Cd in rice is often controlled to greater extent by its bioavailability than its total content in the soil^3^. Therefore, the concentrations of Cd in rice leaves had higher correlation with E-Cd.

**Table 3:** The Pearson’s correlation coefficients between heavy metals concentrations in the soil and the heavy mental concentrations in rice leaves.

		Concentrations in soil			
		Cd	E-Cd	Pb	E-Pb
Concentrations in rice leaves	Cd	0.169	0.649*	—	—
	Pb	—	—	0.222	0.340

^*^means at the 0.05 significance level.

### Relationship of E-HM concentrations against hyperspectral data

**Figure 2:**
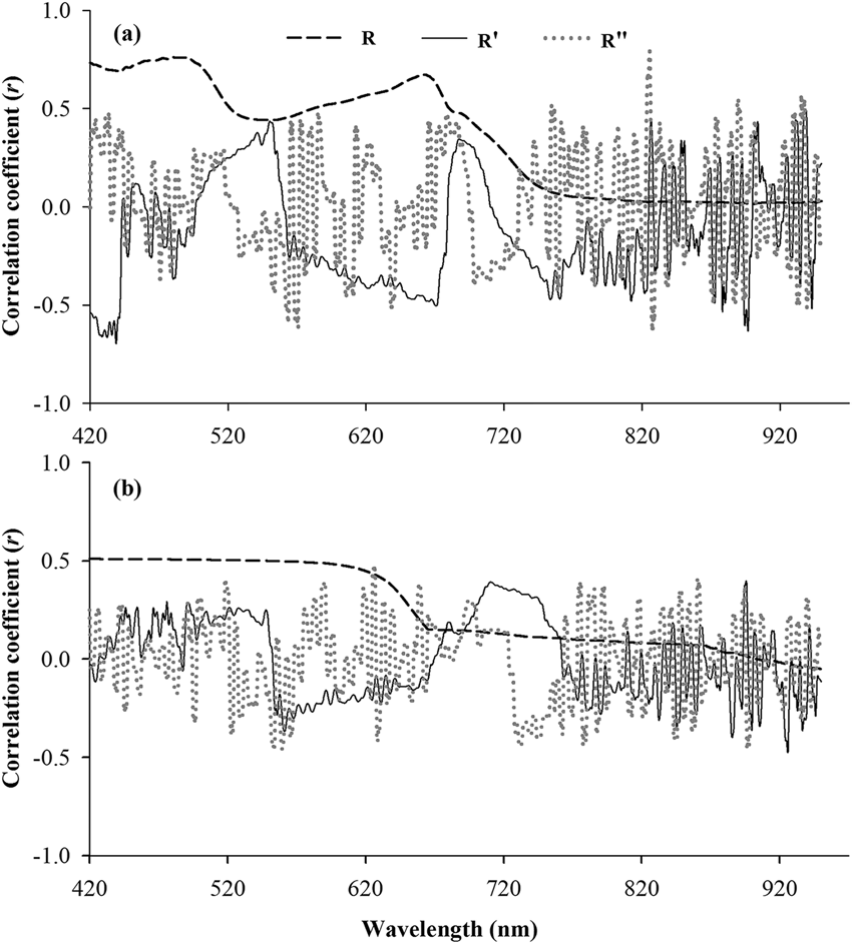
Correlations between processed reflectance (R- raw reflectance; R′- the 1st derivative spectra; R′′- the second derivative spectra) spectra and E-Cd (**a**) and E-Pb (**b**) concentrations in soil from Zhangjiagang city.

The Pearson’s correlation coefficients of the E-HM concentrations and their processed reflectance (R, R′ and R′′) are shown in Fig. 2 and Table 4, and the Pearson’s correlation coefficients between the E-HM concentrations and spectral indices are summarized in Table 5. The wavelengths with significant at *P* < 0.05 indicate that these bands are sensitive to E-HM.

**Table 4:** Correlation analysis between E-HM concentrations and transformations of spectra.

Heavy metal	Types of spectral processing	Maximum positive correlation wave (nm)	Correlation coefficient	Confidence level	Minimum negative correlation wave (nm)	Correlation coefficient	Confidence level
E-Cd	R	480	0.761	**	—	—	—
	R'	939	0.480	*	439	−0.696	**
	R′′	824	0.680	**	569	−0.542	*
E-Pb	R	420	0.511	*	950	−0.050	—
	R'	711	0.391	—	926	−0.462	*
	R′′	625	0.421	*	559	−0.450	*

**means at the 0.01 significance level, *means at the 0.05 significance level.

**Table 5:** The Pearson’s correlation coefficients between the E-HM concentrations and spectral indices.

	NDVI	SR	VOGI	mSR_705_	ARI	WI	PRI2	CRI	NPCI	RVSI
E-Cd	−0.705^**^	−0.411	−0.416	−0.222	−0.269	−0.235	−0.525^*^	−0.665^**^	−0.477^*^	−0.3
E-Pb	0.259	0.191	0.193	0.096	−0.163	0.195	0.002	−0.02	0.1	−0.35

**means at the 0.01 significance level, *means at the 0.05 significance level.

From Table 4, we could see that the maximum positive correlation waves and the minimum negative correlation waves between E-Cd and E-Pb were different. As shown in Fig. 2a, the number of bands associated with E-Cd gradually decreases as the processing progresses. There were 277 bands (in the range of 420–696 nm) of R, 68 bands of R′ and 37 bands of R′′ had significant relationship (*P* < 0.05) with E-Cd concentrations in soil. The correlation bands of R were continuous, while the correlation bands of R′ and R′′ were dispersed. In some literature correlation, the similar relationship between heavy metals and spectral data were shown^51,52^. This indicated that heave metal stress leads to spectral response, but redundant information was contained among the very close spectral bands^53,54^. Pre-processing techniques could remove redundancy information and made some subtle information clear in the spectral in order to improve the subsequent multivariate regression^55^.

While in Fig. 2b, we could see that the trend of the relationships between spectral and E-Pb concentrations was similar to that between spectral and E-Cd concentrations, but whether it in R, R′ or in R′′ correlograms, there correlations coefficient were not reach the 0.01 significance level (Table 4). That may be due to the low concentrations of E-Pb in the agricultural soil, which has not caused obvious stress on rice and has no obvious effect on the leaf spectra.

As shown in Table 5, spectral indices showed a wide range of correlations with the concentrations of E-Cd (−0.705–0.222) and E-Pb (−0.35–0.259). All spectral indices were negatively related to E-Cd concentrations, four of them (NDVI, CRI, PRI2 and NPCI) had significant correlation (P < 0.05) with E-Cd content, while none index had significant correlation with E-Pb concentrations. The four spectral indices associated with E-Cd concentrations were leaf pigment-related indices. The index related to leaves water content (WI) had no significant correlation with E-Cd concentrations. Because Cd can damage the structure of chloroplasts, as manifested by the disturbed shape and the dilation of the thylakoid membranes^56^, so the indices associated with leaf pigment were more susceptible to Cd. However, rice water content is resistant to Cd when the mass fraction of Cd in 2.0–3.0 mg/kg in farmland soil^57^. According to Table 2, the mean content of Cd in agricultural soil was 0.3 mg/kg, which in the region of the resistant. Therefore, the spectral index related to water content had no significant associate with E-Cd concentrations.

### Model development and validation

We selected 386 and 209 variables for the model of E-Cd concentrations and E-Pb concentrations respectively, and the number of the samples was 21, meanwhile, most of the selected variables have strong collinearity, so the PLSR models was very suitable for this study.

**Figure 3:**
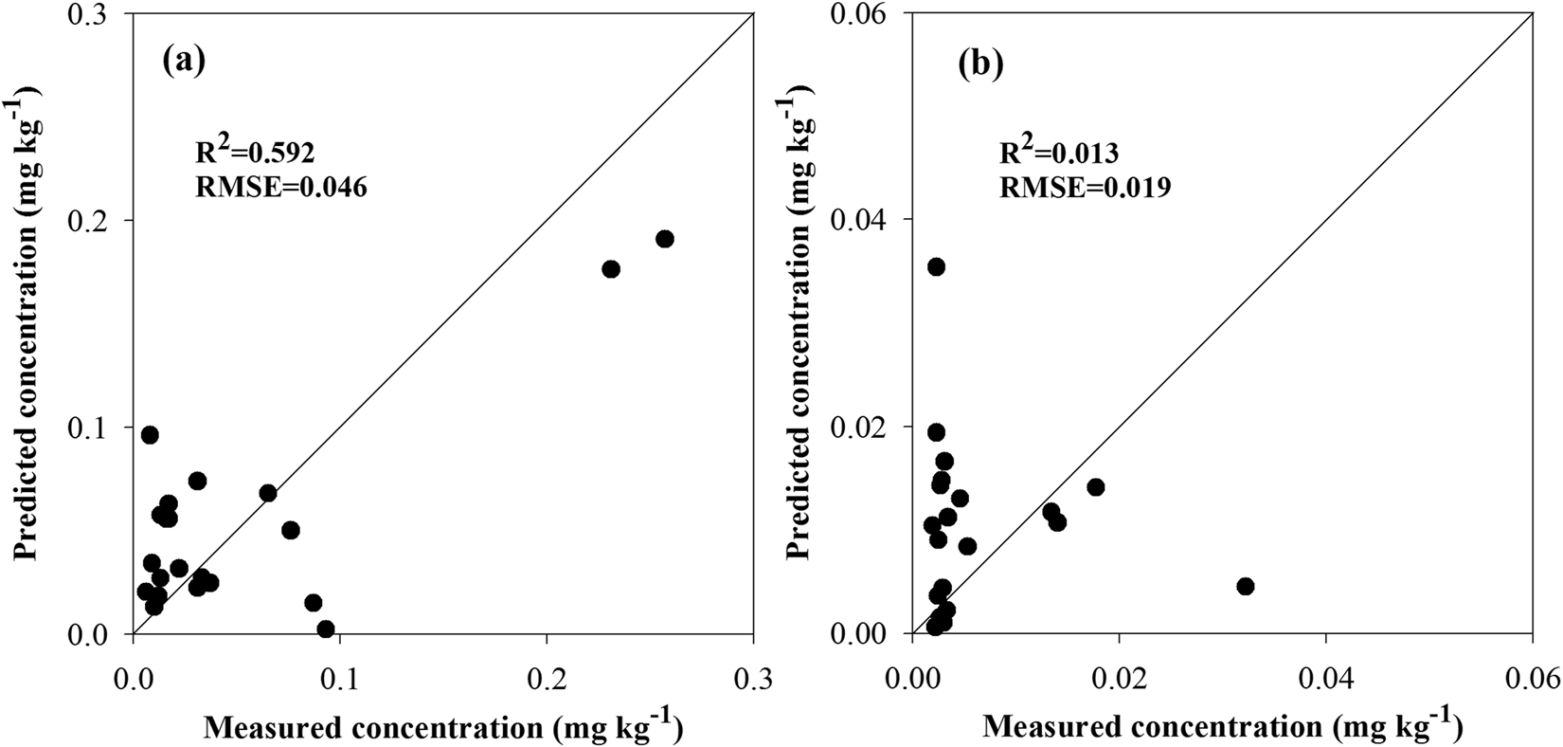
The relationship between measured and predicted E-Cd (**a**) and E-Pb (**b**) concentration in soil based on PLSR models.

The relationship between measured concentrations of E-HM and predicted concentrations of E-HM were presented in Fig. 3. A proper model should have low RMSE and *R*^2^ should be close to 1^7^. It was clear that the PLSR model had the capacity to predict E-Cd content, due to its higher coefficients of determination (*R*^2^ = 0.592) and low RMSE (0.046) (Fig. 3a). While, the prediction of the PLSR for E-Pb concentrations with the RMSE value was 0.019 and *R*^2^ only achieved 0.013, did not show good (Fig. 3b). It is known from the literature that Cd is the best-known toxic heavy metal and it is taken up by the calcium uptake system in plants^58^, while the soil has a higher binding capacity for Pb than for Cd^59^, making Pb less bioavailable. And from Table 2 we also knew that the ratio of the E-Cd concentrations in the total Cd (E-Cd_mean_/Cd_mean_ = 0.051/0.301 = 0.17) was higher than the ratio of E-Pb concentrations in the total Pb (E-Pb_mean_/Pb_mean_ = 0.01/29.193 = 0.0001), so rice may stressed by Cd not by Pb. The accurate of the PLSR model of E-Cd concentrations was not very high, that may contribute to only 21 sampling points were used for model development and validation, which impact on the robustness of the models.

In summary, it was demonstrated that, if the rice was sensitive to E-HM or it was stressed by a certain concentration of E-HM, the PLSR model based on pretreatment reflectance from hyperspectral data of rice leaves had the capability to estimate E-HM concentrations.

## Conclusions

In present study, the concentration of Cd in 19.05% of samples points exceeded the limit set by MEEPRC in agricultural soil of Zhangjiagang city, and the concentration of E-Cd in soil had significant correlation with concentration of Cd in rice leaves. However, due to the low concentration and the low bioavailability of Pb, the concentration of E-Pb in soil had no significant correlation with concentration of Pb in rice leaves.

The raw reflectance had redundant information, and pre-processing techniques could remove redundancy information and made some subtle information clear in the spectral. So the number of bands associated with E-HM gradually decreases as the processing progresses (R > R′ > R′′). The number of bands associated with E-Cd was more than that of E-Pb; the correlation between E-Cd concentrations and spectral data was higher than that between E-Pb concentrations and spectral data. Meanwhile, because of the low concentration of the E-Pb and the Cd resistant of rice water content, there were four indices (NDVI, CRI, PRI2 and NPCI), which related to chlorophyll or pigment were significant correlated with E-Cd concentrations.

The PLSR model had the capacity to estimate E-Cd concentrations in agricultural soil, but cannot estimate E-Pb concentrations in agricultural soil because of the low concentration of E-Pb. So, if the crop was sensitive to E-HM or the crop was stressed by the E-HM, the PLSR model had the capacity to estimate E-HM concentrations in soil.

Using hyperspectral data to evaluate E-HM content in agricultural soil is not affected by soil chemical properties (such as soil pH, organic matter content and soil texture), which can directly reflect the toxicity of heavy metals in soil and has a wider range of applications and a more accurate result compared with the total heavy metals concentration assessment method. This method may provide a new insight to monitoring the E-HM content in agricultural soil. However, the number of samples was too low to use an external validation, so more samples will be collected in the future to improve the predictive performances, and more heavy metals will be estimated to test robustness of the model.

## References

[1] Kumar SR, Agrawal M, Marshall F (2007). Heavy metal contamination of soil and vegetables in suburban areas of Varanasi, India. Ecotoxicology & Environmental Safety.

[2] Stasinos S, Zabetakis I (2013). The uptake of nickel and chromium from irrigation water by potatoes, carrots and onions. Ecotoxicology & Environmental Safety.

[3] Hu Y, Cheng H, Tao S (2016). The challenges and solutions for cadmium-contaminated rice in China: A critical review. Environment International.

[4] Nordberg G, Bernard A, Fierens S, Buchet JP (2002). Low bone density and renal dysfunction following environmental cadmium exposure..

[5] Chen J (2007). Rapid urbanization in china: A real challenge to soil protection and food security. Catena.

[6] Hao X, Zhou D, Wang Y, Shi F, Jiang P (2011). Accumulation of Cu, Zn, Pb, and Cd in edible parts of four commonly grown crops in two contaminated soils. International Journal of Phytoremediation.

[7] Liu M, Liu X, Wu M, Li L, Xiu L (2011). Integrating spectral indices with environmental parameters for estimating heavy metal concentrations in rice using a dynamic fuzzy neural-network model. Computers & Geosciences.

[8] Choe E (2008). Mapping of heavy metal pollution in stream sediments using combined geochemistry, field spectroscopy, and hyperspectral remote sensing: A case study of the rodalquilar mining area, se spain. Remote Sensing of Environment.

[9] Koster M, Reijnders L, van Oost NR, Peijnenburg WJ (2005). Comparison of the method of diffusive gels in thin films with conventional extraction techniques for evaluating zinc accumulation in plants and isopods. Environmental Pollution.

[10] Alexander M (2000). Aging, bioavailability, and overestimation of risk from environmental pollutants. Environmental Science & Technology.

[11] Harmsen J (2007). Measuring bioavailability: From a scientific approach to standard methods. Journal of Environmental Quality.

[12] Tessier A, Campbell PGC, Bisson M (1979). Sequential extraction procedure for the speciation of particulate trace metals. Analytical Chemistry.

[13] Rauret G (1999). Improvement of the BCR three step sequential extraction procedure prior to the certification of new sediment and soil reference materials. Journal of Environmental Monitoring Jem.

[14] Davlson W, Zhang H (1994). *In situ* speciation measurements of trace components in natural waters using thin-film gels. Nature.

[15] Fedotov PS (2012). Extraction and fractionation methods for exposure assessment of trace metals, metalloids, and hazardous organic compounds in terrestrial environments. Critical Reviews in Environmental Science & Technology.

[16] Ettler V, Mihaljevič M, Šebek O, Grygar T (2007). Assessment of single extractions for the determination of mobile forms of metals in highly polluted soils and sediments—analytical and thermodynamic approaches. Analytica Chimica Acta.

[17] Meers ESR (2007). Phytoavailability assessment of heavy metals in soils by single extractions and accumulation by phaseolus vulgaris. Environmental & Experimental Botany.

[18] Houba VJG, Lexmond TM, Novozamsky I, Lee JJvd (1996). State of the art and future developments in soil analysis for bioavailability assessment. Science of The Total Environment.

[19] Novozamsky I, Lexmond TM, Houba VJG (1993). A single extraction procedure of soil for evaluation of uptake of some heavy metals by plants. International Journal of Environmental Analytical Chemistry.

[20] Anjos C, Magalhães MCF, Abreu MM (2012). Metal (Al, Mn, Pb and Zn) soils extractable reagents for available fraction assessment: Comparison using plants, and dry and moist soils from the braçal abandoned lead mine area, portugal. Journal of Geochemical Exploration.

[21] Liu B, Ai S, Zhang W, Huang D, Zhang Y (2017). Assessment of the bioavailability, bioaccessibility and transfer of heavy metals in the soil-grain-human systems near a mining and smelting area in NW China. Science of the Total Environment.

[22] Behrens T, Müller J, Diepenbrock W (2006). Utilization of canopy reflectance to predict properties of oilseed rape (brassica napus l.) and barley (hordeum vulgare l.) during ontogenesis. European Journal of Agronomy.

[23] Zhang T (2011). Using hyperspectral vegetation indices as a proxy to monitor soil salinity. Ecological Indicators.

[24] Zabcic, N., Rivard, B., Ong, C. & Mueller, A. In *Workshop on Hyperspectral Image & Signal Processing: Evolution in Remote Sensing*.

[25] Wang J (2014). Prediction of low heavy metal concentrations in agricultural soils using visible and near-infrared reflectance spectroscopy. Geoderma.

[26] Wu Y (2007). A mechanism study of reflectance spectroscopy for investigating heavy metals in soils. Soil Science Society of America Journal.

[27] Zhao D, Reddy KR, Kakani VG, Read JJ, Carter GA (2003). Corn (zea mays l.) growth, leaf pigment concentration, photosynthesis and leaf hyperspectral reflectance properties as affected by nitrogen supply. Plant & Soil.

[28] Smith KL, Steven MD, Colls JJ (2004). Use of hyperspectral derivative ratios in the red-edge region to identify plant stress responses to gas leaks. Remote Sensing of Environment.

[29] Carter GA, Miller RL (1994). Early detection of plant stress by digital imaging within narrow stress-sensitive wavebands. Remote Sensing of Environment.

[30] Delalieux S (2009). Hyperspectral reflectance and fluorescence imaging to detect scab induced stress in apple leaves. Remote Sensing.

[31] Camoglu G, Demirel K, Genc L (2017). Use of infrared thermography and hyperspectral data to detect effects of water stress on pepper. *Quantitative Infrared Thermography*. Journal.

[32] Schwartz G, Eshel G, Ben-Dor E (2011). Reflectance spectroscopy as a tool for monitoring contaminated soils..

[33] Zhang M, Qin Z, Liu X, Ustin SL (2003). Detection of stress in tomatoes induced by late blight disease in california, USA, using hyperspectral remote sensing. International Journal of Applied Earth Observation & Geoinformation.

[34] Hamzeh S (2013). Estimating salinity stress in sugarcane fields with spaceborne hyperspectral vegetation indices. International Journal of Applied Earth Observation and Geoinformation.

[35] People’s Government of Zhangjiagang City. *Zhangjiagang statistical yearbook of 2017*, http://www.zjg.gov.cn/zfxxgk/075003/075003028/075003028004/moreinfozdly.html (2018).

[36] China Soil Database. *China soil taxonomy database*, http://soil.geodata.cn (1995).

[37] Ran J, Wang D, Wang C, Zhang G, Zhang H (2016). Heavy metal contents, distribution, and prediction in a regional soil-wheat system. Science of the Total Environment.

[38] Zhu, L., Di, S. & Hui, X. Comparison of acid digestion methods for the determination of heavy metals in soil of vegetable lands. *Chinese Agricultural Science Bulletin* (2007).

[39] Gomez C, Lagacherie P, Coulouma G (2008). Continuum removal versus PLSR method for clay and calcium carbonate content estimation from laboratory and airborne hyperspectral measurements. Geoderma.

[40] Tiezhu, S., Yiyun, C., Yaolin, L. & Guofeng, W. Review: Visible and near-infrared reflectance spectroscopy—an alternative for monitoring soil contamination by heavy metals. *Journal of Hazardous Materials*, 166–177 (2014). 10.1016/j.jhazmat.2013.11.059 24361494

[41] Pandit CM, Filippelli GM, Li L (2010). Estimation of heavy-metal contamination in soil using reflectance spectroscopy and partial least-squares regression. International Journal of Remote Sensing.

[42] Wang F, Gao J, Yong Z (2018). Hyperspectral sensing of heavy metals in soil and vegetation: Feasibility and challenges. Isprs Journal of Photogrammetry & Remote Sensing.

[43] Vasques GM, Grunwald S, Sickman JO (2008). Comparison of multivariate methods for inferential modeling of soil carbon using visible/near-infrared spectra. Geoderma.

[44] Shao X, Bian X, Liu J, Zhang M, Cai W (2010). Multivariate calibration methods in near infrared spectroscopic analysis. Analytical Methods.

[45] Liu M, Liu X, Ding W, Wu L (2011). Monitoring stress levels on rice with heavy metal pollution from hyperspectral reflectance data using wavelet-fractal analysis. International Journal of Applied Earth Observation & Geoinformation.

[46] Ministry of Ecology and Environment of the People’s Republic of China. *Soil environmental quality risk control standard for soil contamination of agricultural land*, http://kjs.mee.gov.cn/hjbhbz/bzwb/trhj/trhjzlbz/201807/W020180703592044203183.pdf (2018).

[47] Maiz I, Esnaola MV, Millán E (1997). Evaluation of heavy metal availability in contaminated soils by a short sequential extraction procedure. Science of the Total Environment.

[48] Rennert T, Rinklebe J (2017). Modelling the potential mobility of Cd, Cu, Ni, Pb and Zn in mollic fluvisols. Environmental Geochemistry & Health.

[49] Brümmer, G. W. In *The importance of chemical “speciation” in environmental processes* Vol. 33 (Springer,Berlin, Heidelberg, 1986).

[50] Smith R (2009). S. A critical review of the bioavailability and impacts of heavy metals in municipal solid waste composts compared to sewage sludge. Environment International.

[51] Rathod PH (2015). Spectral changes in the leaves of barley plant due to phytoremediation of metals -results from a pot study. European Journal of Remote Sensing.

[52] Tiezhu S (2014). Monitoring arsenic contamination in agricultural soils with reflectance spectroscopy of rice plants. Environmental Science & Technology.

[53] Demir B, Ertürk S (2008). Phase correlation based redundancy removal in feature weighting band selection for hyperspectral images. International Journal of Remote Sensing.

[54] Held M, Rabe A, Senf C, Linden SVD, Hostert P (2017). Analyzing hyperspectral and hypertemporal data by decoupling feature redundancy and feature relevance. IEEE Geoscience & Remote Sensing Letters.

[55] Rinnan Å, Berg Fvd, Engelsen SB (2009). Review of the most common pre-processing techniques for near-infrared spectra. TrAC Trends in Analytical Chemistry.

[56] Ouzounidou G, Moustakas M, Eleftheriou EP (1997). Physiological and ultrastructural effects of cadmium on wheat (triticum aestivum l.) leaves. Archives of Environmental Contamination & Toxicology.

[57] Guan L, Liu X (2009). Hyperspectral recognition models for physiological ecology characterization of rice in cd pollution stress. Ecology & Environmental Sciences.

[58] Nies DH (1999). Microbial heavy-metal resistance. Applied Microbiology & Biotechnology.

[59] Wieczorek, J., Baran, A., Urbański, K., Mazurek, R. & Klimowicz-Pawlas, A. Assessment of the pollution and ecological risk of lead and cadmium in soils. *Environmental Geochemistry & Health*, 1–18 (2018). 10.1007/s10653-018-0100-5 PMC6280874 29589150

[60] Tucker CJ (1979). Red and photographic infrared linear combinations for monitoring vegetation. Remote Sensing of Environment.

[61] Rouse, J. W. *Monitoring vegetation systems in the great plains with ERTS*. Vol. 1 (NASA SP-351, U.S.Govt. Printing Office, 1974).

[62] Vogelmann JE, Rock BN, Moss DM (1993). Red edge spectral measurements from sugar maple leaves. International Journal of Remote Sensing.

[63] Sims DA, Gamon JA (2002). Relationships between leaf pigment content and spectral reflectance across a wide range of species, leaf structures and developmental stages. Remote Sensing of Environment.

[64] Gitelson AA, Merzlyak MN, Chivkunova OB (2001). Optical properties and nondestructive estimation of anthocyanin content in plant leaves. Photochemistry & Photobiology.

[65] Peñuelas J, Filella I, Biel C, Serrano L, Savé R (2007). The reflectance at the 950–970 nm region as an indicator of plant water status. International Journal of Remote Sensing.

[66] Filella I, Amaro T, Araus JL, Peñuelas J (1996). Relationship between photosynthetic radiation-use efficiency of barley canopies and the photochemical reflectance index (PRI). Physiologia Plantarum.

[67] Gitelson AA, Zur Y, Chivkunova OB, Merzlyak MN (2002). Assessing carotenoid content in plant leaves with reflectance spectroscopy. Photochemistry & Photobiology.

[68] Blackburn GA (1998). Spectral indices for estimating photosynthetic pigment concentrations: A test using senescent tree leaves. International Journal of Remote Sensing.

[69] Merton, R. & Huntington, J. In *Summaries of the Eight JPL Airborne Earth Science Workshop* 299–307 (NASA Jet Propulsion Lab, 1999).

